# Relationship between chronic hypoxia and seizure susceptibility

**DOI:** 10.1111/cns.13942

**Published:** 2022-08-18

**Authors:** YuanHang Xu, QingLi Fan

**Affiliations:** ^1^ Qinghai University Graduate School Xining China; ^2^ Department of Neurology Qinghai Provincial People's Hospital Xining Xining China

**Keywords:** AQP4, brain‐derived neurotrophic factor, cerebral blood flow, chronic hypoxia, glial cells, high altitude, hypothalamus‐pituitary‐adrenal axis, neuroinflammation, neurosteroids, oxidative stress, seizure susceptibility, sodium potassium ATPase, stress, Wnt/β‐catenin

## Abstract

Chronic hypobaric hypoxia in high‐altitude areas is closely related to the occurrence of many neurological diseases. Among these diseases, epilepsy is a common disease of the nervous system that is difficult to diagnose and treat, with a long treatment cycle. As of 2019, there were more than 70 million epilepsy patients worldwide, including 10 million in China. Studies have shown that chronic hypoxia promotes the occurrence and development of epilepsy, and elucidation of the relationship between chronic hypoxia and epilepsy is important for studying the pathogenesis of epilepsy and exploring the potential characteristics of epilepsy and new drug targets for epilepsy. In this article, we review the factors that may cause increased seizure susceptibility in chronic hypoxia and consider the potential relationship between chronic hypobaric hypoxia and seizure susceptibility in high‐altitude areas and prospects surrounding related research in the future.

## Graphical abstract

Chronic hypoxia induces oxidative stress and neuro inflammation as one of the important ways to enhances seizure susceptibility.

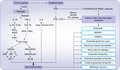

## 1 INTRODUCTION

Chronic hypobaric hypoxia in high‐altitude areas can affect the functions of the heart,^1^ cerebrovascular system, and respiratory system, impair cognition, accelerate the progression of neurodegenerative diseases, seriously affect the normal physiological function of body systems, and even cause systemic diseases.^2^ Qinghai Province is located in northwestern China, with an average elevation of more than 3200 meters. Its natural hypobaric hypoxic environment provides a unique advantage for studying neurological diseases caused by chronic hypoxia. In recent years, with the increase in construction on the Qinghai‐Tibet Plateau and the need for development in the western region of China, a large number of engineers, scholars, and other relevant personnel from low‐altitude areas have entered the plateau, which is likely to cause prominent public health problems with regard to the neurological system in the plateau area of China. Therefore, the study of neurological diseases caused by chronic hypobaric hypoxic environments has broad prospects.

To date, research investigating neurological diseases caused by chronic hypoxia in international academic research has mainly focuses on the fields of cerebrovascular diseases, brain cognitive function, and nervous system degenerative diseases.^3^ However, few studies have investigated the relationship between chronic hypoxia and epilepsy. In addition, the treatment of epilepsy focuses on the control of symptoms after the onset of epilepsy,^4^ and there is a lack of in‐depth studies on the early prevention and treatment of epilepsy and the geographical characteristics of its onset. Acute hypoxia can lead to epileptic seizures, and its clinical manifestations are similar to those of idiopathic epilepsy. However, epileptic seizures associated with acute hypoxia are also often accompanied by pathological changes such as neuronal ischemic necrosis and the formation of partial cortical softening foci. What is the relationship between chronic hypoxia and epilepsy? Previous studies have found that chronic hypoxia can cause various pathological changes in the limbic system and neurons in the brain^5^, ^6^ and that most of these changes may cause increased neuronal excitability. Therefore, chronic hypoxia may be involved in the occurrence and development of epilepsy and increase seizure susceptibility. In this article, by elucidating the common cause of brain tissue changes induced by chronic hypoxia and brain tissue pathological changes that can cause increased neuronal excitability and lower the epileptic seizure threshold, the relationship between chronic hypoxia and seizure susceptibility is theoretically analyzed. The argument that chronic hypoxia causes increased seizure susceptibility is verified, and the relationship between natural hypobaric hypoxia and seizure susceptibility in the Qinghai‐Tibet Plateau is explored. The specific content of this article is as follows.

## 2 CHRONIC HYPOXIA ENHANCES SEIZURE SUSCEPTIBILITY BY ACTIVATING MICROGLIA AND DESTROYING THE NORMAL FUNCTION OF ASTROCYTES AND NEURONS

### 2.1 Chronic hypoxia induces abnormal activation of microglia by inducing oxidative stress and neuroinflammation

#### 2.1.1 The role of microglia in the central nervous system (CNS)

Second to neurons, glial cells exhibit the highest levels of expression in the human body and play many roles in the CNS, such as nutrition, support, and neuro repair.^7^ Glial cells include astrocytes, microglia and oligodendrocytes, microglia are the resident phagocytic cells of the CNS.^8^ Microglia play an important role in the immune function of the CNS. When the CNS is injured by trauma, infection, ischemia or hypoxia, the activity of microglia increases, and microglia participate in the central immune response. Thus, microglia protect the central nervous system and glia and neurons from external damage and play a role in maintaining the normal function of glia and nerve cells and homeostasis within the CNS.^9^

#### 2.1.2 Chronic hypoxia can lead to excessive release of reactive oxygen species, central oxidative stress, secondary neuroinflammation, and abnormal activation of microglia

**FIGURE 1:**
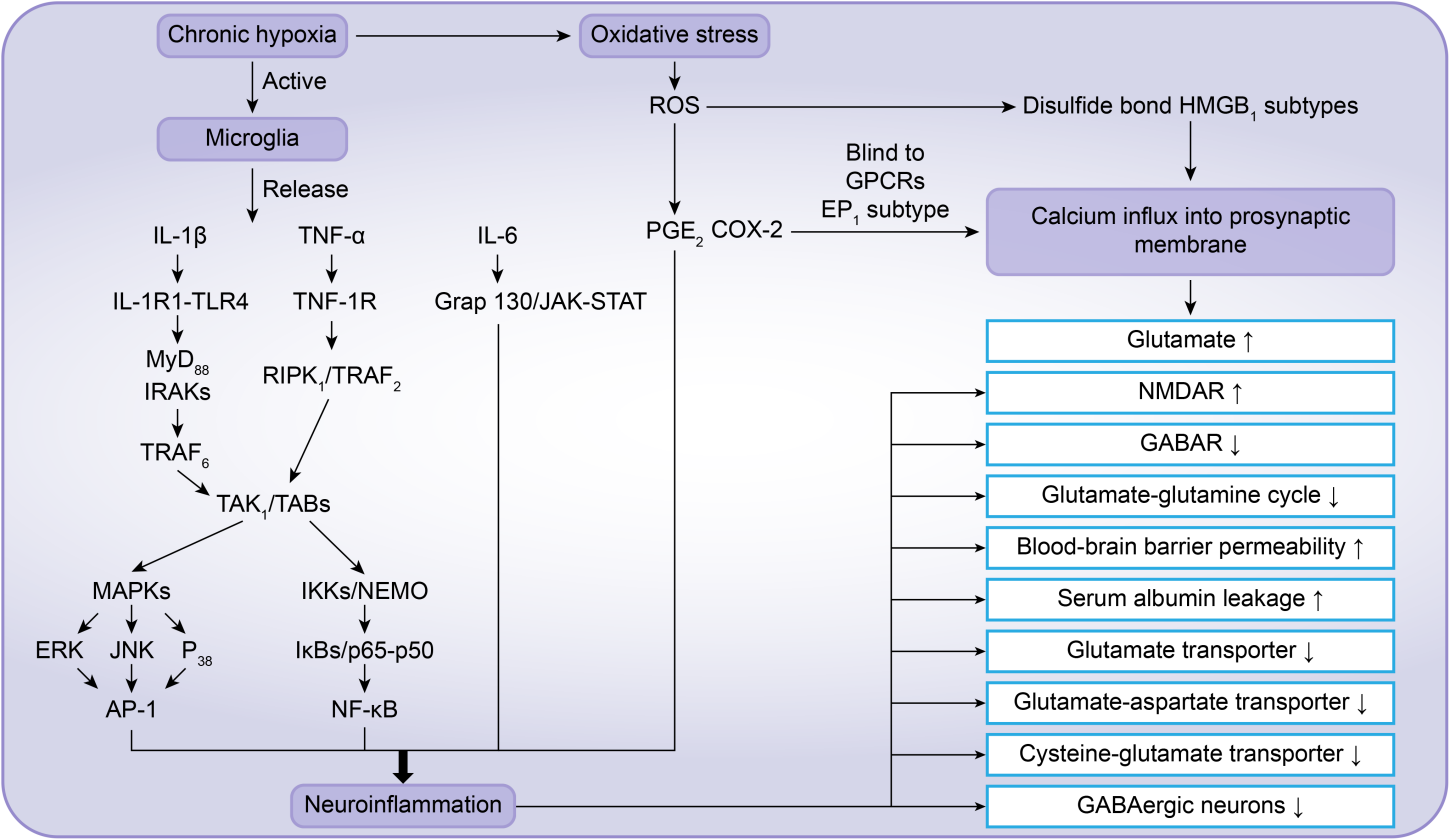
Chronic hypoxia can cause oxidative stress and ROS release and induce neuroinflammation. Chronic hypoxia can also induce neuroinflammation by activating microglia and the release of inflammatory factors. The released inflammatory factors activate downstream pathways to affect neurotransmitter transporters, blood–brain barrier permeability, and neurotransmitters and their receptors, thereby directly or indirectly increasing seizure susceptibility. Notably, the oxidative stress product ROS and its product PGE2 link oxidative stress with neuroinflammation, enabling oxidative stress and neuroinflammation to act synergistically in promoting neuronal excitotoxicity

**FIGURE 2:**
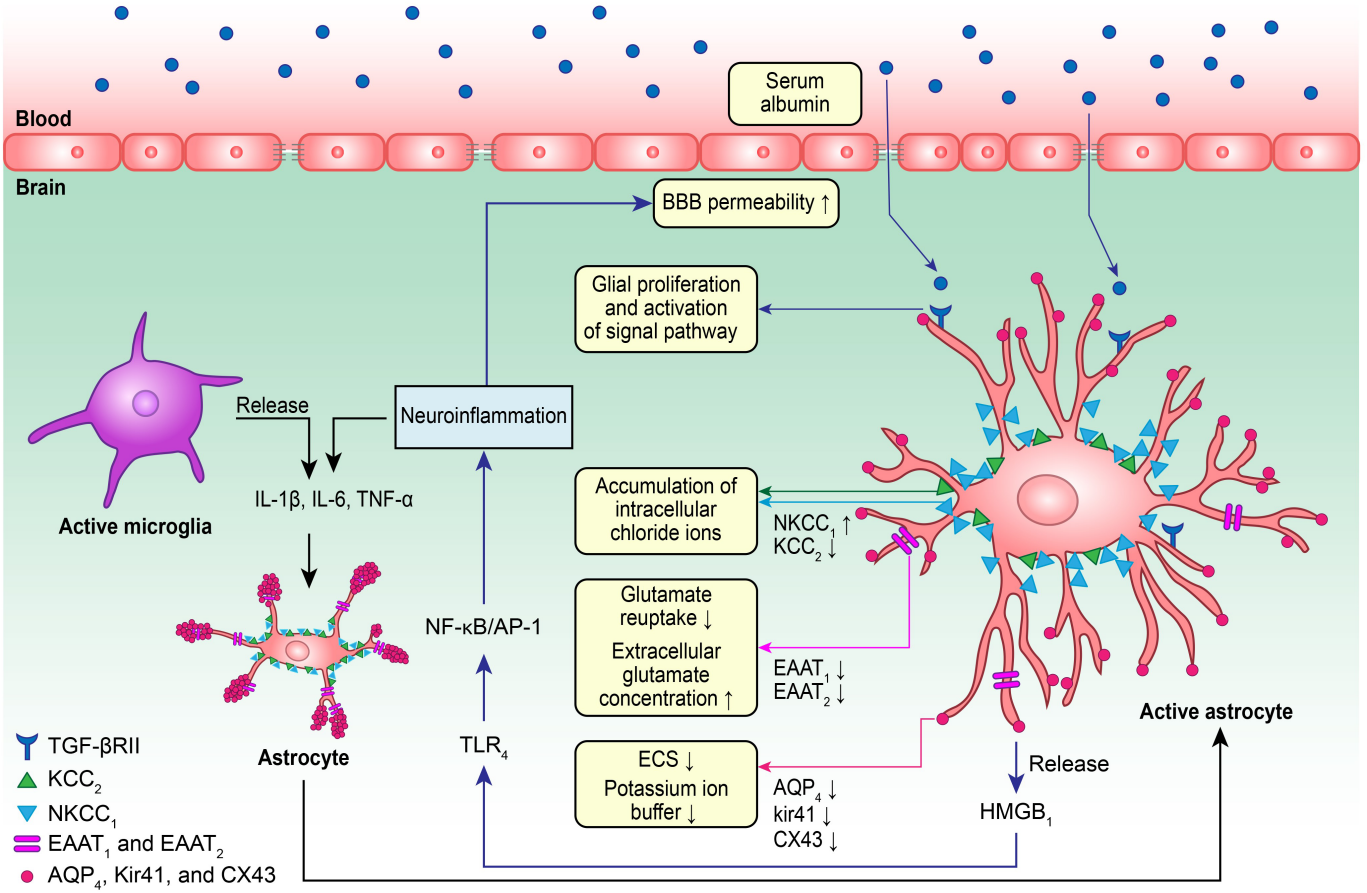
Neuroinflammation secondary to chronic hypoxia can increase the permeability of the blood–brain barrier and promote albumin leakage. Albumin entering the central nervous system can bind to transforming growth factor β 2 receptor and activate the smad2 pathway, resulting in abnormal astrocyte activation and proliferation. The abnormal activation and proliferation of astrocytes are accompanied by decreases in physiological function, such as increased expression of NKCC1 (cyan arrow), decreased expression of KCC2 (green arrow), decreased expression of EAAT1 and EAAT2 (purple arrow), and decreased expression of AQP4, Kir4.1 and CX43 (red arrow), and trigger a series of changes that could enhance neuroexcitotoxicity (corresponding to the yellow box). Abnormally activated and proliferated astrocytes can release HMGB1, which activates the downstream TLR4 pathway to induce neuroinflammation and the release of inflammatory cytokines. The inflammatory cytokines subsequently act on astrocytes and increase blood–brain barrier permeability (blue arrow), thus forming a vicious circle

Chronic hypoxia can cause abnormal energy metabolism in cells and increase the production and release of reactive oxygen species (ROS) from mitochondria through transcription and phosphorylation of NADPH oxidase (NOX) and increasing the expression of inducible nitric oxide synthase (iNOX), thus increasing the level of oxidative stress in the CNS.^10^, ^11^ High levels of reactive oxygen species can increase the expression of cyclooxygenase 2 (COX‐2) and its main product prostaglandin 2 (PGE2)^12^ and then activate microglia and induce neuroinflammation (Figure 1). PGE2 has a separate effect on neurons, which is mainly reflected in the high affinity between PGE2 and EP1 subtypes of G protein‐coupled receptors. PGE2 can increase calcium influx and glutamate release in the presynaptic membrane by binding to EP1^11^ (Figure 1). Oxidative stress and neuroinflammation show a complementary relationship, and high mobility group protein 1 (HMGB1) is an important carrier between oxidative stress and neuroinflammation. Oxidative stress can induce glial cells and neurons to release a variety of damage‐related molecular patterns (DAMPs), including HMGB1, which is important.^13^ HMGB1 normally shows a completely reduced state. Only oxidative HMGB1 can bind to Toll‐like receptor 4 (TLR4),^14^ activate downstream pathways, and promote the translocation of nuclear factor kappa B (NF‐κB) to the nucleus of microglia, causing the release of inflammatory factors and mediating neuroinflammation^13^, ^15^(Figure 2). When the body is in a state of chronic hypoxia, subsequent oxidative stress can cause a large amount of ROS release, and ROS can not only promote HMGB1 oxidation through disulfide bonds to produce disulfide bond HMGB1 subtypes (Figure 1) but also enhance the stability of HMGB1 disulfide bond subtypes^14^; thus, HMGB1 is the link between oxidative stress and neuroinflammation. Oxidative stress secondary to chronic hypoxia can produce ROS, and the resulting ROS can stabilize HMGB1 disulfide subtypes and induce neuroinflammation; thus ROS and HMGB1 reflect the relationship between oxidative stress and neuroinflammation, as they promote each other and cascade amplification.^14^ This relationship promotes the continuous progression of chronic hypoxia‐mediated neurotoxicity in the CNS (HMGB1 can also have a separate effect on neurons: HMGB1 mediates calcium influx of NR2B‐NMDA receptor subunits through tyrosine protein kinase Src, thus promoting calcium overload and glutamate release^16^). The relationship between HMGB1 and seizure susceptibility has been confirmed by animal experiments. Fu, L., et al found that HMGB1 injection into the ventricles of mice could shorten the latency of chemical kindling and increase the grade of seizures in mice,^17^ thus confirming that HMGB1 can increase seizure susceptibility in rodents. Therefore, chronic hypoxia may enhance neuronal excitotoxicity and seizure susceptibility by increasing the level of central oxidative stress and neuroinflammation, activating microglia, and promoting the release of proinflammatory mediators and excitatory neurotransmitters.^10^, ^12^, ^18^

#### 2.1.3 Chronic hypoxia can cause chronic neuroinflammation and abnormal activation of microglia, and persistent susceptibility to spreading depolarization

**FIGURE 3:**
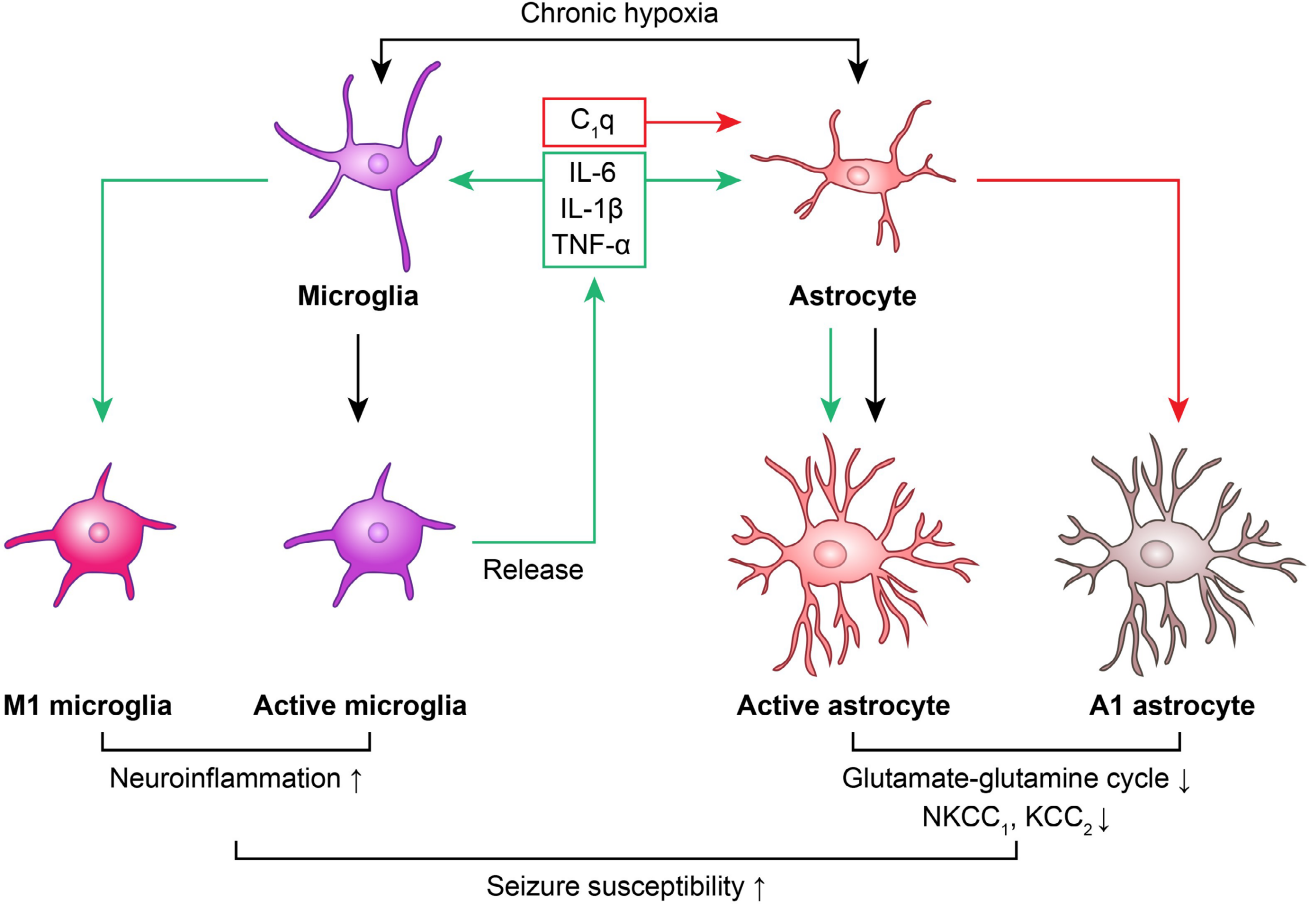
Chronic hypoxia can activate astrocytes and microglia (black arrow). Activated microglia release inflammatory factors, complements and other inflammatory mediators. These inflammatory factors act on microglia to transform them into M1 microglia (green arrow), and the inflammatory factors and complements act on astrocytes to transform them into A1 astrocytes (green and red arrows), thus forming a vicious circle. Activated microglia, astrocytes, and their corresponding M1 and A1 glial subtypes can enhance seizure susceptibility in various ways

As immune cells of the CNS, microglia are activated when the body is in a chronic hypoxic environment, leading to increased expression of ionized calcium binding adapter molecule 1 (IBA‐1), a marker of microglial activation, and microglial release of proinflammatory mediators such as interleukin 1β (IL‐1β), interleukin 6 (IL‐6), and tumor necrosis factor α (TNFα).^13^, ^19^, ^20^, ^21^, ^22^ IL‐1β and TNF‐α released by microglia combine with interleukin 1 receptor type 1 (IL‐1R1) and tumor necrosis factor‐αR1 (TNFR1) to jointly activate downstream nuclear factor κB (NF‐κB) and activating protein 1 (AP‐1).^13^ IL‐6 activates the Grp130/JAK–STAT pathway,^13^ and the two pathways together promote further expression of downstream inflammatory factors (Figure 1). Among the above inflammatory factors, IL‐1β is the main factor inducing chronic neuroinflammation. IL‐1β activates the IL‐1R1‐TLR4 signaling pathway by binding to IL‐1R1, which can decrease the expression of the GABA receptor and increase the expression of the NMDA receptor in neurons and glial cells.^23^ Lai, A.Y. et al found that activation of IL‐1R1‐TLR4 signaling enhances the function of the NMDA receptor in cultured hippocampal neurons through a non‐transcriptional mechanism, thus promoting NMDA‐induced calcium influx and the release of excitatory neurotransmitters such as glutamate.^24^ Accordingly, activation of this signaling pathway can destroy the dynamic balance of various neurotransmitters and the excitability of neurons. Galic, M.A. found that treating rodent hippocampal slices with IL‐1β induces NMDA receptor‐mediated calcium influx in neurons via tyrosine kinase phosphorylation of the NR2B subunit of the NMDA receptor.^25^ Moreover, high levels of proinflammatory mediators in CNS inflammation will subsequently act on microglia to promote their continuous activation and transformation into M1 microglia^8^ (Figure 3). As a result, neurons and glial cells in the CNS are continuously affected by chronic neuroinflammation.

Chronic neuritis has multiple effects on the CNS. The main effects include inhibiting the function of neurotransmitter transporters (including glutamate transporter, glutamate aspartate transporter, and cysteine glutamate transporter), improving the concentration of extracellular glutamate^13^ and the permeability of the blood–brain barrier, and aggravating protein leakage^11^, ^26^ (See Part 1.2.2 below for details) (Figure 1). Chronic neuroinflammation also changes neuronal voltage and ligand‐gated ion channel activity, resulting in a reduction in GABAergic neurons^27^, ^28^ and excitatory‐associated synaptogenesis (reducing the expression of the GABAA‐type receptor on the plasma membrane, enhancing the function of NMDA receptors in neurons and inducing calcium overload secondary to calcium influx mediated by phosphorylation of the NMDA receptor NR2B subunit^29^), a decrease in the glutamate‐glutamine cycle rate,^13^ regulation of intracellular and extracellular ion concentrations, and activation of neurotransmitter function by astrocytes^30^, ^31^ (Figure 1).

Activated microglia can not only further induce neuroinflammation but also increase neuronal excitability by maintaining susceptibility to spreading depolarization (SD), a transient electrical signal that can spread slowly in the gray matter and cortex of the brain. Because SD often occurs during the process of migraine aura and epilepsy, it is often regarded as a potentially harmful event that destroys the normal excitability of neurons.^32^ Microglia are closely related to the occurrence of SD, as activated microglia can reduce the SD threshold and maintain SD susceptibility by regulating calcium transduction signaling, thus increasing the incidence of SD. The occurrence of SD is often accompanied by destruction of the transmembrane ion gradient and increases in extracellular potassium ion and glutamate concentrations in neurons and glial cells.^32^ Consequently, microglial activation‐induced SD may be accompanied by abnormal enhancement of neuronal excitability and participate in the occurrence and development of epilepsy to some extent.^33^ The above perspective has been confirmed in some studies. Through the use of colony stimulating factor 1 receptor (CSF1R) kinase inhibitor to reduce the number of microglia, Varga, D.P., et al. confirmed that a significantly increased threshold was required for recurrent SD.^32^ A study conducted by Dreier, J.P. Mantegazza, M. and S. Cestèles revealed a link between SD and epilepsy.^33^ Lin, C.H., et al. found that SD could induce epileptic activity in CA1 pyramidal neurons of mouse hippocampal slices, suggesting the involvement of SD induced by microglia activation in the increase in seizure susceptibility.^34^

Therefore, chronic hypoxia can activate microglia to maintain SD susceptibility, induce neuroinflammation, release cytokines and chemokines, and activate downstream signaling pathways to establish a self‐amplification cascade to improve neuronal excitability and increase seizure susceptibility.

### 2.2 Chronic hypoxia induces abnormal activation and proliferation of astrocytes by inducing neuroinflammation and improving blood–brain barrier permeability

#### 2.2.1 The role of astrocytes in the CNS

In the central nervous system, the main physiological functions of astrocytes include the regulation of neurotransmitter receptor activity, the production and transport of neurotransmitters, the maintenance of ion, energy substrate, and osmotic pressure homeostasis,^21^ the promotion of neurogenesis and gliogenesis, and the constitution of the blood–brain barrier and maintenance of its integrity. Glial fibrillary acidic protein (GFAP) is an important skeletal protein synthesized by astrocytes and is a characteristic marker of astrocytes. GFAP expression increases during the proliferation and activation of astrocytes. Astrocytes are a double‐edged sword. The physiological proliferation of astrocytes helps maintain homeostasis in the central nervous system environment and protects neurons (compensatory effect), while abnormally proliferating astrocytes can affect the excitability of central neurons and homeostasis in the central nervous system (decompensation effect).

#### 2.2.2 Chronic hypoxia causes abnormal activation and proliferation of astrocytes

**FIGURE 4:**
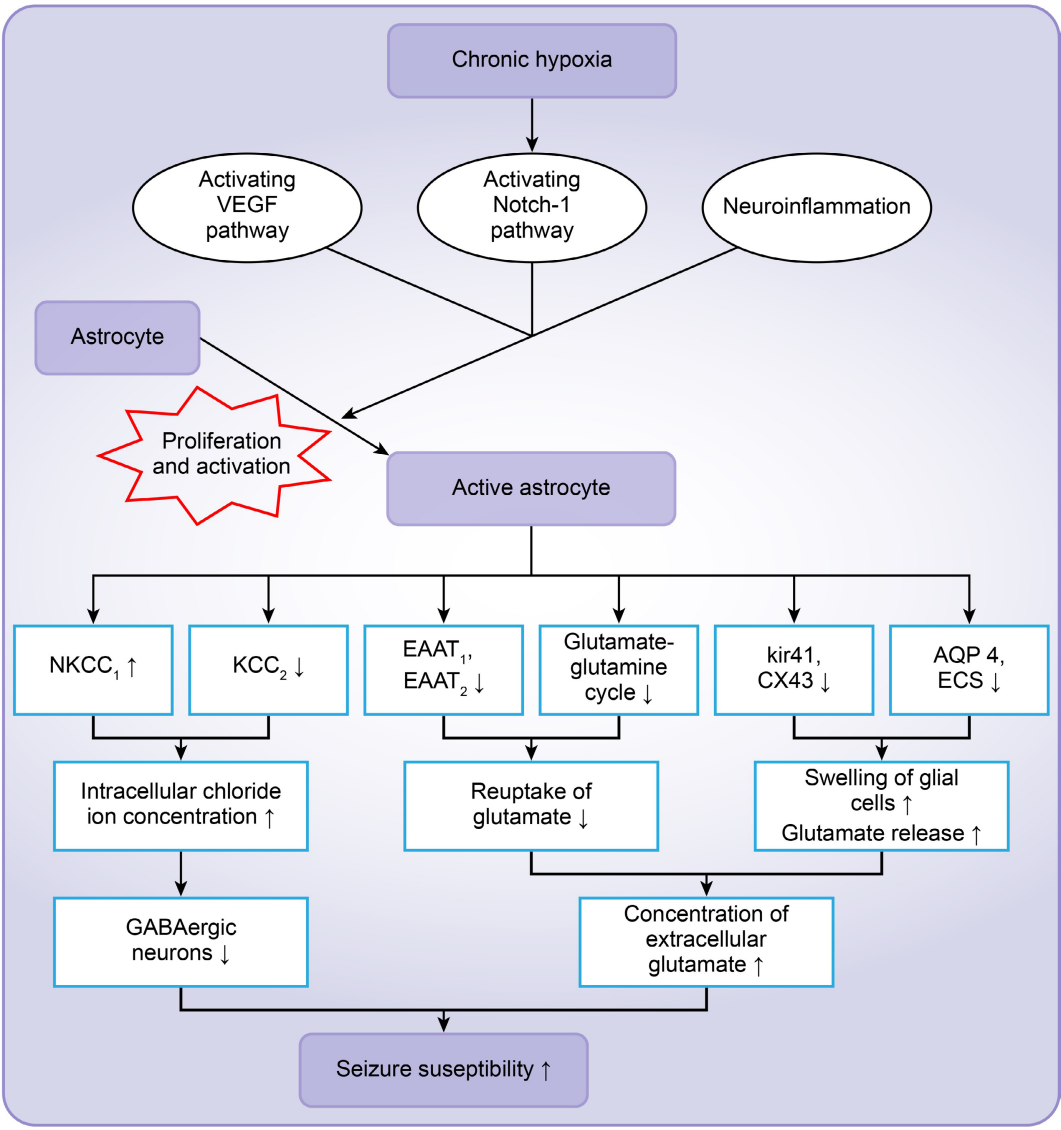
Chronic hypoxia and secondary neuroinflammation can activate astrocytes and decrease their normal physiological function, with changes in the expression of excitatory amino acid transporters, ion channels, connexin, and AQP4. These changes enhance seizure susceptibility by promoting intracellular chloride deposition, reducing glutamate reuptake, promoting glutamate release secondary to cell swelling, increasing extracellular glutamate concentrations, and reducing GABAergic neuronal inhibition

Chronic hypoxia may promote the abnormal proliferation and activation of astrocytes by activating VEGF or Notch‐1 pathways^35^, ^36^, ^37^, ^38^, ^39^ (Figure 4). Neuroinflammation secondary to chronic hypoxia can also abnormally activate astrocytes and affect their normal physiological functions.^12^ In addition, the complement C1q released in neuritis merges with astrocyte nodules and converts them into neurotoxic type A1 astrocytes^21^ (Figure 3). Abnormally proliferating and activated reactive astrocytes and type A1 astrocytes will have a series of abnormal functional changes (such changes are mainly decompensated‐related changes), mainly including decreased expression of glutamate transporter EAAT2 and glutamate aspartate transporter EAAT1,^40^ a decreased glutamate‐glutamine cycle rate,^27^ decreased expression of inward rectifier potassium channel 4.1 (Kir4.1) and AQP4 colocated at the cell terminal foot,^11^ cell swelling, increased glutamate release (induced by cell swelling),^41^ increased expression of sodium potassium chloride symporter (NKCC1),^42^, ^43^ decreased extracellular space (ECS),^41^, ^44^ and decreased expression of potassium chloride symporter (KCC2)^27^, ^42^, ^44^ and connexin 43 (Cx43)^11^, ^27^ (Figure 4). The above changes can improve neuronal excitability and increase seizure susceptibility in various manners, as follows:

Abnormal activation and proliferation of astrocytes caused by chronic hypoxia can enhance neuronal excitability at the level of neurotransmitters. Decreased expression of EAAT1 and EAAT2 in reactive astrocytes and a decreased glutamate‐glutamine cycle rate may reduce glutamate reuptake, while reduced ECS and cell swelling secondary to decreased AQP4 expression may increase glutamate release and increase the concentration of extracellular glutamate and other excitatory neurotransmitters^13^, ^45^, ^46^, ^47^ (AQP4 expression in the early stage of hypoxia is believed to increase to compensate for the swelling of the cell body and the reduction in ECS.^48^ With prolonged hypoxia, the expression of AQP4 and its colocated proteins Kir4.1 and CX43, which maintain cell tight junctions, decreases due to decompensation^46^, ^49^). The increase in the glutamate concentration corresponds to a decrease in GABAergic neuronal activity, and the change in NKCC1 and KCC2 expression in reactive astrocytes mediates this effect. Since the inhibitory effect of GABAergic neurons is achieved through the transmembrane distribution gradient of chloride ions, a low concentration of intracellular chloride plays an important role in maintaining the inhibitory effect of GABA,^27^, ^28^ while chronic hypoxia promotes the entry of chloride into glial cells and inhibits the accumulation of intracellular chloride ions by increasing NKCC1 expression and reducing KCC2 expression (see part 2.2 below). Therefore, chronic hypoxia reduces the activity of GABAergic neurons by increasing the concentration of intracellular chloride ions and then reduces their excitatory inhibition.^27^, ^50^

**FIGURE 5:**
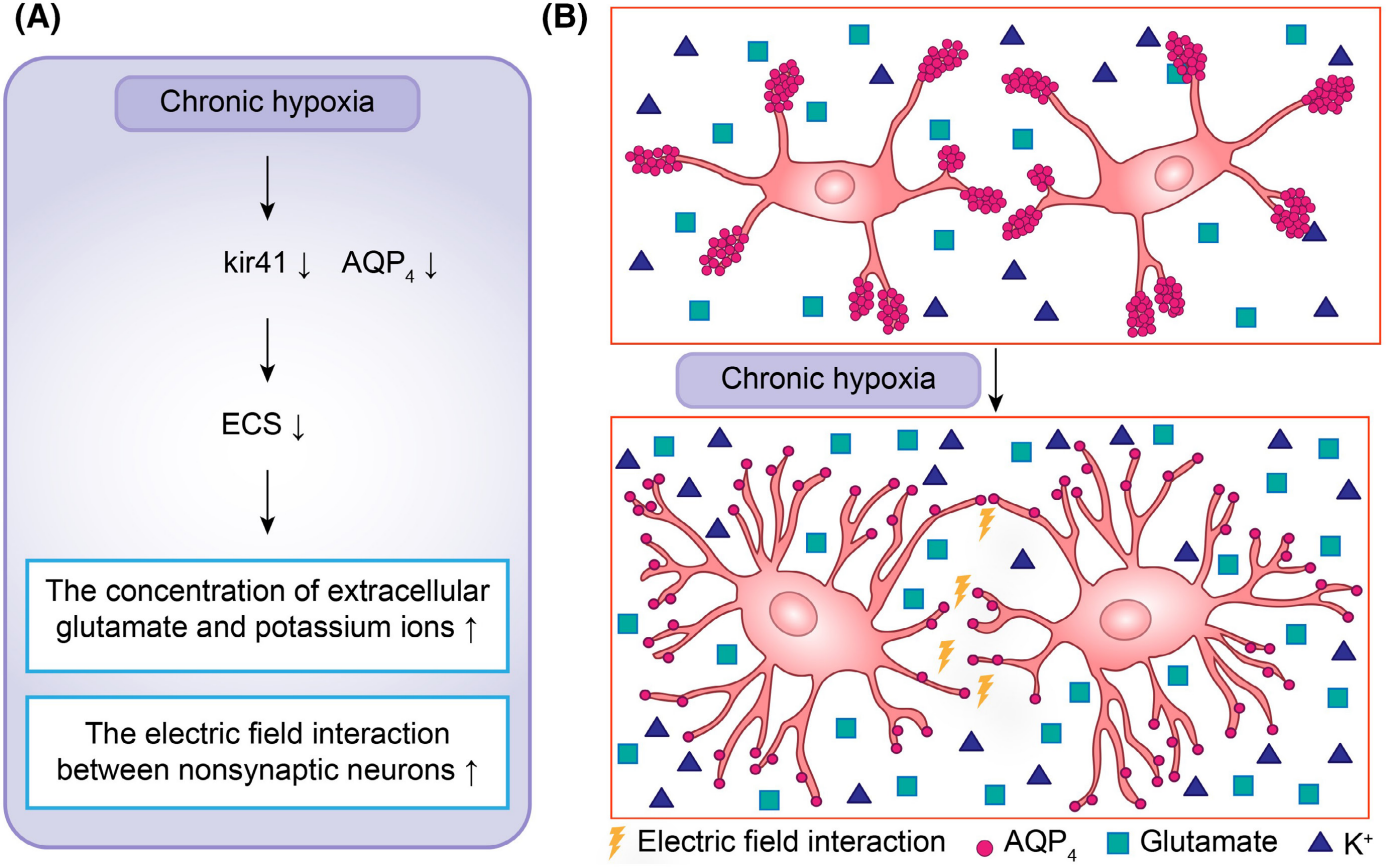
Chronic hypoxia can affect the physiological function of astrocytes through abnormal activation of astrocytes, resulting in decreases in Kir4.1 and AQP4 in the end feet of astrocytes. The reduced expression of Kir4.1 AQP4 and astrocyte swelling can increase the concentrations of glutamate and potassium ions in the ECS while reducing the distance between synapses, thereby elevating seizure susceptibility by lowering neuronal excitability thresholds and increasing the interaction of non‐synaptic electric field forces between cells

Chronic hypoxia can not only enhance neuronal excitability at the neurotransmitter level but also reduce the neuronal excitability threshold at the ionic level. The decreased expression of Kir4.1 and CX43 in reactive astrocytes can cause extracellular potassium deposition and potassium buffering between neurons and glial cells. With the reduction in ECS, the increase in extracellular potassium concentration and the neurotoxic effect of ion deposition become increasingly serious, while the increase in the extracellular potassium concentration can lead to a decrease in the absolute resting potential and the distance between resting potential and threshold potential.^51^ Then, seizure susceptibility is increased due to a reduced excitation threshold of neurons. In addition, ECS reduction can cause neurons to be close to each other at the spatial level and then enhance the electric field interaction between non‐synaptic neurons^52^ (Figure 5), resulting in an increase in neuronal excitability.

Therefore, chronic hypoxia can enhance seizure susceptibility by activating astrocytes, enhancing neuronal excitability, and reducing the seizure threshold at the levels of neurotransmitters and ions.

#### 2.2.3 Chronic hypoxia induces the proliferation and activation of astrocytes by increasing the permeability of the blood–brain barrier

The blood–brain barrier connects the human CNS with the circulatory system and regulates the flow of metabolites, drugs, and nutrients into and out of the CNS through its own neurovascular units; therefore, the blood–brain barrier plays an irreplaceable role in maintaining the homeostasis of the CNS and the normal excitability of neurons. In the CNS, cerebral vascular endothelial cells, astrocyte terminal feet, pericytes, and connexins are all involved in the formation of neurovascular units in the blood–brain barrier.^53^ However, neuroinflammation secondary to chronic hypoxia and the release of inflammatory factors and chemokines associated with M1 pro‐inflammatory microglia can damage the function of astrocytes, reduce the expression of tight junction proteins, and damage endothelial cells and neurovascular units. In addition, oxidative stress secondary to chronic hypoxia can increase the activity of iNOS and promote the production of NO. NO combines with superoxide anion (O2‐) to form peroxynitrite (ONOO‐), which causes extensive damage to cerebral microvessels via excessive consumption of glutathione and induction of lipid peroxidation.^54^ Chronic hypoxia damages the physiological function of the blood–brain barrier by increasing its permeability and triggers a series of neurotoxic reactions through the above‐mentioned side effects of neuroinflammation and oxidative stress. The increase in blood–brain barrier permeability can cause peripheral serum protein leakage, which is mainly manifested by the entry of peripheral albumin into the blood–brain barrier and astrocyte binding to TGF‐β receptor II, causing secondary glial proliferation and activating the smad2 signaling pathway.^11^, ^13^, ^26^, ^53^ A series of subsequent gene transcriptional changes significantly reduce the normal physiological function of astrocytes (including downregulation of Kir4.1, AQP4, EAAT1, EAAT2, and CX43), which is mainly characterized by impaired extracellular potassium and glutamate buffers^11^, ^13^ (Figure 2). In addition, activation of TGF‐β signaling can also induce abnormal neurogenesis, increase the production of excitatory synapses of neurons, and promote astrocytes to release proinflammatory factors such as HMGB1, and the released proinflammatory factors such as HMGB1 activate their downstream pathways and trigger intracellular signal cascades, thus causing excitotoxic damage to neurons and other surrounding cells^11^, ^30^ (Figure 2).

Therefore, chronic hypoxia can induce abnormal activation and proliferation of astrocytes by increasing the permeability of the blood–brain barrier and enhance neuronal excitotoxicity and seizure susceptibility.

## 3 CHRONIC HYPOXIA INCREASES SEIZURE SUSCEPTIBILITY BY CHANGING THE EXPRESSION OF SODIUM POTASSIUM ATP ENZYMES, NKCC1, AND KCC2

### 3.1 Chronic hypoxia can decrease the activity of sodium and potassium ATP enzymes in brain tissue

Three kinds of α subunit NA‐K‐ATPases are expressed in the CNS, and the α2 subunit is specifically expressed in glial cells, while the α3 subunit is specifically expressed in neurons (especially GABA neurons).^55^ NA‐K‐ATPase is the main unit of ATP consumption in brain tissue. NA‐K‐ATPase consumes ATP to maintain the potassium and sodium concentrations inside and outside neurons and glial cells and regulate calcium transmembrane transport and neurotransmitter release and reuptake^56^; thus, NA‐K‐ATPase plays an important role in maintaining the normal excitability of neurons and the dynamic balance of neurotransmitters.

Chronic hypoxia may have a negative effect on NA‐K‐ATPase activity by inhibiting energy metabolism, reducing ATP production, increasing ATP consumption and ROS production, and inducing mutations in genes encoding NA‐K‐ATPase‐related subunits (NA‐K‐ATPase α 3 subunits in neurons encoded by the ATP1A3 gene^57^).^58^ Farhat, E., et al. found that exposure to chronic hypoxia for 3 months reduced NA‐K‐ATPase activity in goldfish brains by 40%.^56^ Silva, L.F.A., et al. confirmed that ROS inhibits NA‐K‐ATPase activity by oxidizing the sulfhydryl (sh) groups and changing the fluidity of the nerve cell membrane.^58^

The decrease in NA‐K‐ATPase activity may increase seizure susceptibility by reducing the excitability threshold of neurons and increasing the concentration of extracellular glutamate and calcium overload. Under physiological conditions, NA‐K‐ATPase can maintain the resting potential and repolarization of neurons by transporting three sodium ions out of the neurons and sending two potassium ions into the neurons. When the activity of NA‐K‐ATPase decreases, the increase in the intracellular sodium concentration can lead to a decrease in the absolute resting potential, the distance between resting potential and threshold potential, and the excitability threshold of neurons to some extent.^51^ Moreover, the sodium concentration gradient maintained by NA‐K‐ATPase is the driving force for glial cells to reuptake glutamate. The decrease in NA‐K‐ATPase activity can reduce glutamate reuptake and increase extracellular glutamate concentrations.^57^ Karus, C., et al. confirmed that when the intracellular sodium concentration increase to 12 Mmol, the driving force for glutamate uptake decreases by approximately 20%.^59^ In addition, the increase in the intracellular sodium concentration can lead to a secondary increase in sodium and calcium exchange, while calcium influx can lead to excitatory synaptic glutamate release and neurotoxicity and neuronal death secondary to calcium overload,^57^ further increasing seizure susceptibility. Freitas, M.L.D., et al. confirmed that the NA‐K‐ATPase activator DRRSAb reduced glutamate release in pilocarpine‐kindled mouse brain slices in vitro.^60^ Funck, V.R., et al. found that DRRSAb injection into the hippocampus of mice delayed pentylenetetrazol‐induced seizures and reduced their seizure susceptibility.^61^ Krishnan, G.P., et al. demonstrated that the sodium and potassium ATP enzyme blocker STDN can increase seizure susceptibility and prolong seizure times in experimental animals.^62^ In addition to pharmacological evidence, the study of gene mutant mice with NA‐K‐ATPase damage also supports the view that a decrease in NA‐K‐ATPase activity can enhance the excitability of the nervous system.^61^ Therefore, chronic hypoxia may decrease the excitability threshold of neurons and increase the concentration of extracellular glutamate and calcium overload by reducing the activity of sodium and potassium ATP enzymes in brain tissue.

### 3.2 Chronic hypoxia increases seizure susceptibility by increasing NKCC1 expression and decreasing KCC2 expression

Both NKCC1 and KCC2 are cationic‐chloride cotransporters, which realize chloride ion transport across the cell membrane through the electrochemical gradient produced by NA‐K‐ATPase.^44^ NKCC1 and KCC2 mediate the influx and outflow of chloride, respectively, to maintain the low concentration of chloride in neurons and glial cells. Because the GABAA receptor is a ligand‐gated chloride channel and the inhibitory activity of GABAergic neuron transmission is controlled by the concentration gradient of transmembrane chloride ions, a low intracellular chloride concentration plays a leading role in the inhibitory effect of GABAergic neurons.^27^ Thus, the normal function of NKCC1 and KCC2 is very important for maintaining the concentration of the chloride ion transmembrane distribution and the normal excitability of neurons.

Chronic hypoxia can reduce the expression of NKCC1.^43^ In addition, chronic hypoxia may trigger an intracellular signal cascade response by activating the BDNF–TrkB and pro‐BDNF‐p75NTR pathways by increasing the secretion of brain‐derived neurotrophic factor (BDNF) and forebrain‐derived neurotrophic factor (pro‐BDNF), resulting in increased NKCC1 expression and decreased KCC2 expression.^27^, ^44^ As a result, the increase in the NKCC1‐dominated chloride influx effect and the decrease in the KCC2‐dominated chloride outflow effect led to chloride deposition, while intracellular chloride deposition reduced the inhibitory effect mediated by GABAergic neurons, resulting in increased excitability of neurons and increased seizure susceptibility^63^ (Figure 4). In addition, chloride ion accumulation in cells can cause glial and nerve cell swelling, and the increase in NKCC1 activity can also lead to a series of excitotoxic injuries by increasing glutamate release and decreasing reuptake induced by cell swelling.^42^

In addition to activating the BDNF‐TrkB and pro‐BDNF‐p75NTR pathways, chronic hypoxia‐induced glial cell activation, neuroinflammation, and oxidative stress can also affect the expression of cation‐chloride cotransporters.^11^, ^27^ In addition, the above‐mentioned effects of chronic hypoxia abnormally activate NMDA receptors by increasing the concentration of extracellular glutamate, and the abnormal increase in NMDA receptor activity can lead to calcium influx into cells and trigger activation of protein phosphatase 1 (PP1) and subsequent KCC2 dephosphorylation, further leading to a decrease in KCC2 membrane expression and intracellular chloride deposition and thus reducing the neuroinhibitory activity of GABA to increase neuronal excitability.^44^, ^64^ Terraneo, L. and M. Samaja reported that BDNF expression increased significantly under hypoxia.^5^ Boulenguez, P., et al. confirmed that BDNF downregulates KCC2 and increases seizure susceptibility through its combination with TrkB.^65^ Sharma, D. and VonDran, M.W. also reported that mild intermittent hypoxia can increase pro‐BDNF expression in the rodent hippocampus, and the combination of pro‐BDNF and p750NTR can also downregulate KCC2 expression.^66^, ^67^ Riffault, B., et al. reported that the increase in BDNF and pro‐BDNF leads to downregulation of KCC2, which in turn leads to the loss of effective depolarization inhibition of GABA signals and overexcitation of neurons.^68^

These results suggest that chronic hypoxia may change the activity of NKCC1 and KCC2 through glial cell activation, neuroinflammation, oxidative stress, and increased pro‐BDNF/BDNF activity and then enhance neuronal excitotoxicity and seizure susceptibility by increasing the extracellular glutamate concentration, inducing chloride deposition and calcium overload, and reducing the inhibitory effect of GABA neurons.

## 4 CHRONIC HYPOXIA MAY ACT AS A SOURCE OF CHRONIC STRESS TO INCREASE SEIZURE SUSCEPTIBILITY BY INHIBITING NEUROSTEROID SECRETION AND ACTIVATING THE HYPOTHALAMUS‐PITUITARY‐ADRENAL AXIS (HPA) AND WNT/Β CATENIN PATHWAY TO REGULATE NEUROGENESIS

### 4.1 Chronic hypoxia may act as a source of chronic stress and stress to increase seizure susceptibility by inhibiting neurosteroid secretion and activating the HPA axis to regulate neurogenesis

As social animals, human beings are constantly faced with various types of stress, although different people face different types of stress. However, a long‐term hypobaric hypoxic environment at high altitude and uncomfortable physiological effects, such as sleep deficiency, temperature differences between day and night, altered gastrointestinal function and activity, and cold, are affecting a large number of people around the world as sources of stress.^68^ When we focus on people who have been affected by hypobaric hypoxia for a long time, studying the relationship between persistent hypoxic pressure and neuronal excitability is of unique significance.

Neurosteroids refer to the steroids synthesized in brain neurons, and their ab initio synthesis may occur in the brain. Neurosteroids mainly include allopregnanolone, tetrahydrodeoxyepiandrosterone, and androstenol. Since the main target of endogenous neurosteroids is the GABA receptor, neurosteroids are believed to have a wide range of effects on regulating the inhibitory effect of GABA neurons in the brain.^70^ Neurosteroids at physiological concentrations can help maintain the stability of neuronal excitability; therefore, they are regarded as convulsion inhibitors.^28^ The main product of the HPA axis is cortisol, which exists in the peripheral nervous system, but because of the lipophilic characteristics of cortisol, cortisol can still have an effect on the CNS. Academic circles believe that long‐term increases in cortisol levels have an adverse effect on the stability of the CNS; therefore, it is regarded as a convulsant.^71^

Both neurosteroids and the HPA axis can be changed under exposure to a variety of pressures and stressors, including chronic hypoxia, and changes in both can affect the excitability of neurons in the CNS. Van Campen, J.S., et al. confirmed that chronic hypoxia can activate the HPA axis and increase the cortisol level in the human body.^71^ Maguire, J. suggested that various chronic stressors may reduce the neuroinhibitory effect of GABA by reducing neurosteroid secretion^28^; thus, the change in neurosteroid secretion and activation of the HPA axis can be regarded as the response of the human body to many types of chronic stressors, including chronic hypoxia. Chronic hypoxia affects a certain number of people worldwide. Long‐term hypoxia will activate the HPA axis and increase cortisol secretion while reducing the secretion level of neurosteroids. Since hypoxia can change neurosteroid secretion and HPA activity as a source of chronic pressure and stress and the relationship between neurosteroid secretion and HPA activity and epilepsy has been confirmed, we hypothesized that hypoxia, as a chronic stressor, enhances seizure susceptibility in humans by changing neurosteroid secretion and HPA activity. How does hypoxic pressure, as a chronic stressor, induce changes in neurosteroids and HPA to enhance seizure susceptibility? How much theoretical basis can we provide for our hypotheses?

By consulting a large amount of data, we found that hypoxia has an anticonvulsant effect as an acute stressor, which is thought to be caused by the increased expression of pressure‐induced neurosteroids, while hypoxia, as a chronic stressor, may increase neuronal excitability by reducing the expression of neurosteroids and inhibiting the inhibitory effect of GABA neurons. Maguire, J. and J.A. Salpekar suggested that chronic stress is a cause of epilepsy.^72^ Maguire, J. found that stress‐related changes in seizure susceptibility and neuronal excitability are mainly attributed to weakening of the inhibitory effect of GABA.^28^ Because the GABAA receptor is one of the main molecular targets of neurosteroids, hypoxia, as a chronic stressor, may enhance neuronal excitability and seizure susceptibility by inhibiting neurosteroid secretion.

Studies have found that adrenocorticotropin‐releasing hormone seems to be a kind of convulsant, and activation of the HPA axis may increase the excitability of neurons by reducing EAAT2 expression and increasing the concentration of extracellular glutamate.^73^ In addition, activation of the HPA axis may have long‐term consequences by changing the neuroendocrine stress response and the structure and function of the brain.^71^ Chronic stress, including hypoxia, can cause long‐term changes in the HPA axis, and such changes in the HPA axis (as mentioned earlier, the main change caused by the HPA axis is an increase in CRH and cortisol secretion) may lead to a decrease in frontal lobe and hippocampal activity and neuronal dendritic atrophy in these areas, which is accompanied by decreased expression of EAAT2 and inhibition of GABA neurogenesis in the hippocampal dentate gyrus. Therefore, chronic stress may increase seizure susceptibility by activating the HPA axis and promoting CRH and cortisol secretion.

In addition, neuronal excitability mediated by chronic stress can be increased through several mechanisms; for example, chronic stress may increase seizure susceptibility by increasing peripheral sulfide neurosteroids (such as DHEAS and PS, mainly regarded as convulsants), inhibiting the activity of GABA neurons, and enhancing the activity of NMDA receptors^70^ or by reducing the binding sites of neurosteroids and GABAA receptors in the cerebral cortex.^74^ Therefore, chronic hypoxia as a stressor may increase seizure susceptibility in many ways, mainly by changing the normal activity of neurosteroids and the HPA axis to inhibit GABA neurogenesis, weakening the inhibitory effect of GABAergic neurons, reducing EAAT2 expression, increasing the extracellular glutamate concentration, and affecting synaptic transmission and hippocampal nerve remodeling, among other effects.

### 4.2 Chronic hypoxia may increase seizure susceptibility by activating Wnt‐β‐catenin to regulate neurogenesis

The Wnt/β‐catenin signaling pathway is responsible for regulating the development of the CNS, adult synaptic plasticity, and adult neurogenesis. The Wnt signaling pathway consists of three intracellular pathways: the classical Wnt/β catenin pathway, planar cellular polarity pathway, and calcium pathway.^75^ This review focuses on the possible effects of chronic hypoxia on the classical Wnt/β catenin pathway and downstream target gene transcription on seizure susceptibility.

Chronic hypoxia may regulate neurogenesis by activating the Wnt/β‐catenin pathway and enhance seizure susceptibility. Under physiological conditions, the Wnt/β‐catenin signaling pathway is initiated by the binding of Wnt ligand to the receptor Frizzled and coreceptors (such as low‐density lipoprotein receptor‐related protein 5). When Wnt ligand binds to Frizzled receptor, intracellular alopecia protein (DVL) is phosphorylated and enhances the stability of β‐catenin. Stable β‐catenin translocates to the nucleus and binds to T cytokines (Tcf) and lymphokines (LEF) to activate the transcription of target genes downstream of Wnt.^76^ Mazumdar, J., et al. confirmed that chronic hypoxia can activate the Wnt‐β catenin signaling pathway by inducing the binding of HIF‐1α to nuclear Lef1 and TCF1 genes and activate the Wnt−/β‐catenin signaling pathway through the classical pathway mentioned above.^77^ Varela‐Nallar, L., et al. also showed that chronic hypoxia could increase the expression of Wnt/β‐catenin in the hippocampus.^76^

Activation of the Wnt/β‐catenin signaling pathway may increase seizure susceptibility. Yang, J., et al. reported that overexpression of β‐catenin may contribute to increased seizure susceptibility.^78^ Hodges, S.L. and J.N. Lugo suggested that activation of the Wnt/β‐catenin signaling pathway helps enhance seizure susceptibility.^75^ How does activation of the Wnt/β‐catenin signaling pathway enhance seizure susceptibility? Hodges, S.L. and J.N. Lugo suggested that β‐catenin overexpression in astrocytes can inhibit neurogenesis and promote neuronal death to mediate neurotoxicity.^75^ To some extent, hypoxia may induce neurotoxicity by activating the Wnt/β‐catenin signaling pathway, regulating neurogenesis, and reducing the number of inhibitory intermediate neurons (such as GABA neurons) and their synaptic connections, thus increasing seizure susceptibility (Table 1).

**TABLE 1:** Mechanisms by which different factors affect epilepsy susceptibility in chronic hypoxia conditions

Cytokine and/or pathways	seizure susceptibility‐related functions	Changes under chronic hypoxia	Possible mechanisms that cause elevated seizure susceptibility	Refs
TNF‐α	Mediates neurotoxicity as an inflammatory factor	↑	Activates astrocytes and microglia, induces neuroinflammation, inhibits glutamate uptake, promotes glutamate release, increases blood–brain barrier permeability, alters neuronal voltage and ligand‐gated ion channel activity, and reduces GABA inhibitory activity of neuron and neuron excitation threshold, increase glutamatergic neuron activity, etc. (induce neuroinflammation and its secondary neurotoxicity)	13, 19, 20, 21, 22
IL‐1β(IL‐1R1‐TLR4)	Mediates neurotoxicity as an inflammatory factor	↑	Activates microglia, induces neuroinflammation, inhibits glutamate uptake, promotes glutamate release, increases blood–brain barrier permeability, alters neuronal voltage and ligand‐gated ion channel activity, reduces inhibitory neurons, increases excitatory related synaptogenesis, etc. (induction of neuroinflammation and its secondary neurotoxicity) Cytokine regulated expression of GABA receptor in neurons and glial cells decreased and expression of NMDA receptor increased (decreased excitability inhibition of GABA neurons) Tyrosine protein kinase Src mediates NR2B‐NMDA receptor subunit phosphorylation induces calcium influx and glutamate release (promotes glutamate release)	13, 19, 20, 21, 22
IL‐6(IL‐6‐Grp130/JAK–STAT)	Mediates neurotoxicity as an inflammatory factor	↑	Activate astrocytes and microglia, induce neuronal inflammation, inhibit glutamate uptake, promote glutamate release, increase blood–brain barrier permeability, alters neuronal voltage and ligand‐gated ion channel activity, decrease GABAergic neuronal inhibitory activity and neuronal excitation threshold, increase glutamatergic neuronal activity, etc. (induce neuroinflammation and its secondary neurotoxic reaction)	13, 19, 20, 21, 22
HMGB1(HMGB1‐TLR4)	The combination of oxidative stress and neuroinflammation can further induce neuroinflammation.	↑	Oxidative stress is associated with neuroinflammation to induce neuroinflammation by activating TOLL‐like receptor 4 and its downstream pathway and promoting the translocation of NF‐ κ B to the nucleus of microglia. Activate astrocytes and microglia and induce inhibition of glutamate uptake, promote glutamate release, increase blood–brain barrier permeability, alters neuronal voltage and ligand‐gated ion channel activity, reduce inhibitory neurons, enhance excitability‐related synaptogenesis and other neuroinflammatory neurotoxic reactions (neuroinflammation and secondary neurotoxicity) NR2B‐NMDA receptor subunit phosphorylation mediated by tyrosine protein kinase Src induces calcium influx, which promotes glutamate release secondary to calcium influx (enhanced glutamate release).	14, 15, 16
ROS	The main products of oxidative stress can further induce nerve inflammation.	↑	Oxidative stress induces neuroinflammation, then activates microglia, inhibits glutamate uptake, promotes glutamate release, increases blood–brain barrier permeability, alters neuronal voltage and ligand‐gated ion channel activity, reduces inhibitory neurons, increases excitatory related synaptogenesis, etc. (induced neurotoxicity) Promote the oxidation of HMGB1 through disulfide bond to produce disulfide bond HMGB1 subtype, and also improve the stability of HMGB1 disulfide bond subtype (stabilize HMGB1 disulfide bond subtype, contribute to the occurrence, and development of neuroinflammation)	10, 11
NKCC1	Promote the entry of chloride ions into neurons	↑	The increase in NKCC1 expression leads to the increase in extracellular chloride ion into the cell, and the accumulation of intracellular chloride ion leads to the swelling of astrocytes, which leads to the increase in glutamate release and the decrease in glutamate reuptake (increased extracellular glutamate concentration) Low concentration of intracellular chloride ion mainly mediates the inhibitory effect of GABAergic neurons, while the increased expression of NKCC1 can promote the entry of chloride ion into glial cells and inhibit the outflow of chloride ion out of glial cells, thus causing intracellular chloride ion deposition and reducing the activity and excitatory inhibitory effect of GABAergic neurons (reducing the excitatory inhibitory effect of GABAergic neurons).	39, 40
KCC2	Promote chloride ion out of neurons	↓	The decrease in KCC2 expression leads to the decrease in chloride output cells, the accumulation of intracellular chloride ions in astrocytes and neurons, the release of glutamate and the increase in glutamate concentration (increased extracellular glutamate concentration). Low concentration of intracellular chloride ion mainly mediates the inhibitory effect of GABAergic neurons, while the decrease in KCC2 expression can inhibit chloride ion outflow from glial cells, which leads to intracellular chloride ion deposition, decrease the activity and excitability inhibition effect of GABAergic neurons (reduce the excitability inhibition effect of GABA neurons).	41, 60
Kir4.1	Buffer extracellular potassium ion	↓	The decrease in Kir4.1 expression leads to the decrease in extracellular potassium buffer capacity and the increase in extracellular potassium concentration. The increase in extracellular potassium concentration can reduce the absolute value of neuronal resting potential and shorten the distance between resting potential and threshold potential, thus reducing the excitability threshold of neurons (reducing excitability threshold).	11, 43, 46
ECS	Provide a buffer environment for extracellular neurotransmitters and ions	↓	The decrease in ECS leads to the increase in extracellular potassium concentration. The increase in extracellular potassium concentration can reduce the absolute value of resting potential, shorten the distance between resting potential and threshold potential, and decrease the excitability threshold of neurons (decrease excitability threshold). The decrease in ECS can make neurons close to each other at the spatial level, thus enhancing the electric field interaction between non‐synaptic neurons (increasing non‐synaptic electric field force interaction).	38, 49
AQP4	Regulate the cross‐membrane flow of extracellular and extracellular water and regulate cell volume	↓	Decreased expression of AQP4 leads to swelling of glial cells, decrease in ECS, increase in potassium ion and glutamate concentration in ECS (increase in non‐synaptic electric field interaction between neurons, decrease in excitability threshold, increase in extracellular glutamate concentration). Cell swelling effect increases glutamate release (promotes glutamate release)	11, 43
Wnt/β‐catenine	Regulation of neuronal system development and neurogenesis	↑	Activate Wnt/ β‐catenin pathway to regulate neurogenesis (reduce neurogenesis of GABAergic neurons and decrease excitatory inhibitory activity of GABAergic neurons)	71, 72, 73
NA‐K‐ATPase	Maintain the resting state of neurons, maintain the potential difference between intracellular and extracellular sodium and potassium ions, and provide ion driving force for glutamate reuptake.	↓	The decrease in Na‐K‐ATPase activity can increase the concentration of sodium ion in glial cells and neurons, reduce the potential difference between resting potential and action potential, and reduce the excitation threshold of neurons (decreased excitability threshold). The sodium concentration gradient maintained by Na‐K‐ATPase is the driving force for glial cells to reuptake glutamate, so decreased Na‐K‐ATPase activity can reduce glutamate reuptake by astrocytes and increase extracellular glutamate concentration (increase extracellular glutamate concentration). The increase in intracellular sodium concentration caused by the decrease in Na‐K‐ATPase activity can lead to the secondary increase in sodium and calcium exchange, and the secondary calcium influx can lead to the increase in glutamate release from excitatory synapses and neuronal death caused by calcium overload (calcium overload increases glutamate release and neuronal death).	48, 52, 53, 54, 55
Pro‐BDNF/BDNF–TrkB	Regulation of KCC2 expression	↑	The increased expression of pro‐BDNF and BDNF can lead to the decrease in KCC2 expression, which in turn leads to the decrease in chloride outflow and chloride deposition, while the accumulation of chloride ions in glial cells and neurons can weaken the excitation inhibition of GABAergic neurons (decreased the excitation inhibition activity of GABAergic neurons). Chloride deposition caused by increased expression of pro‐BDNF and BDNF can cause swelling of glial cells and neurons, resulting in a series of excitotoxic damage by increasing the release of glutamate and decreased reuptake caused by cell swelling (increased extracellular glutamate concentration).	27, 41, 61, 62, 63, 64
neurosteroids	Anticonvulsant	↓	Decreased secretion of neurosteroids attenuated the excitatory inhibition of GABAergic neurons (decreased the excitatory inhibition of GABAergic neurons).	28, 66, 70
cortisol	Promote convulsion	↑	Increased cortisol secretion can inhibit the neurogenesis of GABAergic neurons (reduce the neurogenesis of GABAergic neurons and inhibit the excitatory inhibitory activity of GABAergic neurons).	67, 69
CX43	Potassium ion and neurotransmitter buffer	↓	The decrease in CX43 expression leads to the decrease in extracellular potassium buffer capacity, and the increase in extracellular potassium concentration can reduce the absolute value of resting potential and shorten the distance between resting potential and threshold potential, thus reducing the excitability threshold of neurons (reducing excitability threshold).	11, 27, 43, 46
EAAT1/EAAT2	Regulating the concentration of excitatory neurotransmitters	↓	Decreased expression of EAAT1 and EAAT2 resulted in reduced glutamate reuptake (reduced glutamate reuptake and increased extracellular glutamate concentration).	13, 37
blood–brain barrier permeability	Maintain the homeostasis of the central nervous system	↑	The increased permeability of the blood–brain barrier causes peripheral albumin to enter the blood–brain barrier and bind to astrocyte TGF‐ β RII, which causes glial proliferation and activation of Smad2 signal pathway, decreases the normal physiological function of astrocytes (mainly characterized by down‐regulated expression of KIR4.1, AQP4, EAT1, EAT2 and CX43), causes extracellular potassium deposition and glutamate buffering impairment (increased extracellular glutamate concentration). Activation of TGF‐ β RII can induce abnormal neurogenesis, increase the formation of excitatory synapses of neurons, promote astrocytes to release HMGB1 and activate intracellular signal cascade response, thus cause excitatory damage to neurons and surrounding cells (enhance the excitatory activity of glutamatergic neurons, induce neuronal inflammation and secondary neurotoxicity).	30, 50
Cox‐2	Oxidative stress products can further induce neuroinflammation.	↑	Increased COX‐2 secretion can activate microglia and cause neuroinflammation, which in turn abnormally activates astrocytes and destroys their physiological functions (neuroinflammation and secondary neurotoxicity).	11, 12
PGE2	Oxidative stress products can further induce neuroinflammation.	↑	Increased PGE‐2 secretion can activate microglia and cause neuroinflammation, which in turn abnormally activates astrocytes and destroys their physiological functions (neuroinflammation and secondary neurotoxicity). The binding of PGE‐2 to G protein coupled receptor EP1 subtype results in increased calcium influx and glutamate release from presynaptic membrane(increase glutamate release).	11, 12

Abbreviations: AQP4, Aquaporin 4; BDNF, Brain‐derived neurotrophic factor; Cox‐2, Cyclooxygenase‐2; CX43, connexin 43; EAAT1, Excitatory Amino Acid Transporter 1; EAAT2, Excitatory Amino Acid Transporter 2; ECS, extracellular space; HMGB1, high mobility group protein 1; IL‐1β, interleukin 1β; IL‐6, interleukin 6; KCC2, K‐Cl co‐transporter 2; Kir4.1, inward rectifier K+ channels 4.1; NA‐K‐ATPase, Na+, K+ stimulated ATPase; NKCC1, Na+‐dependent chloride transporter 1; PGE2, Prostaglandin E2; ROS, reactive oxygen species; TNF‐α, tumor necrosis factor α.

## 5 CHRONIC HYPOXIA MAY INCREASE SEIZURE SUSCEPTIBILITY BY REDUCING CEREBRAL BLOOD FLOW

Cerebral blood flow can reflect the metabolism of brain tissue and plays an important role in maintaining the normal physiological function of brain tissue. When the human body is exposed to acute hypoxia, to compensate for the decrease in the partial pressure of oxygen and the increase in the partial pressure of carbon dioxide, cerebral blood flow can be increased by mobilizing certain receptors and promoting the release of vasodilators to meet the normal metabolic needs of the body. With prolonged hypoxia time, the cerebral blood flow will gradually decrease under the effects of an increase in hematocrit, an increase in the blood viscosity coefficient, a change in autonomic nerve activity, and the vasoconstrictor factor released by the vascular endothelium.^79^ Cramer, N.P., et al. showed that after exposure to hypobaric hypoxia for 6 months, the cerebral blood flow of C57BL6/J mice was lower than that of the normoxic control group; this change was primarily attributable to increases in hematocrit and the blood viscosity coefficient.^20^ Liu et al. showed that Tibetan individuals under chronic hypoxia intervention had lower cerebral blood flow at high altitude than at low altitude.^80^ Hayward, N.M., et al. demonstrated that seizure susceptibility is markedly increased in patients with craniocerebral injury. In addition to glial proliferation and the formation of epileptic foci, the decrease in cerebral blood flow is also an important factor; that is, the decrease in cerebral blood flow is involved in the occurrence and development of epilepsy after craniocerebral injury.^81^ In addition, Wu, J., et al. confirmed that cerebral blood flow decreased to varying degrees in patients Sturge–Weber syndrome with cortical calcification, and the degree of the decrease in cerebral blood flow was correlated with epileptic load and seizure severity.^82^ Clinical studies found decreased blood flow and calcification in the hippocampus or cortex in patients with epilepsy. Therefore, the above results verify that the decrease in cerebral blood flow secondary to chronic hypoxia may be related to the increase in seizure susceptibility.

In fact, as the duration of hypoxia increases, the duration of cerebral blood flow in different parts of the brain changes to varying degrees and in opposing ways (that is, under a fixed duration of hypoxia, blood flow increases to one extent in one part of the brain but decreases to a different extent in another part of the brain). According to the neurovascular coupling theory, the increase in neuronal excitability requires cerebral vascular dilatation to meet the huge oxygen and energy consumption demand of brain tissue.^83^ Lim, H.K., et al. also confirmed that vasoconstriction often occurs in pre‐seizure and interphase, while patients generally show cerebral vascular dilatation and increased cerebral blood flow during seizures,^84^ which forms an interesting paradox with the study described in the first half of this section. The cause of this paradox merits further exploration. Consequently, the effect of chronic hypoxia on cerebral blood flow cannot be simply defined as increasing or decreasing, and the specific mechanism of the effect of cerebral blood flow changes on seizure susceptibility remains unclear. Although the research conducted to date has not confirmed the mechanism responsible for the decrease in cerebral blood flow and the relationship between the decrease in cerebral blood flow and seizure susceptibility in Tibetan people at high altitude, the theory concerning the effect of changes in cerebral blood flow secondary to hypoxia on neuronal excitability has been confirmed in related studies.^85^ Therefore, it is important to continue researching the effects of chronic hypoxia and high‐altitude hypobaric hypoxia environments on cerebral blood flow and seizure susceptibility, and it may be helpful to further explore the pathogenesis of epilepsy.

## 6 CONCLUSIONS AND PROSPECTS

In summary, chronic hypoxia not only induce activation and proliferation of microglia and astrocytes through oxidative stress and neuroinflammation but also induce neuronal excitotoxicity by inhibiting the activity of sodium and potassium ATP, increasing the permeability of the blood–brain barrier, changing the expression of ion transporters, and reducing cerebral blood flow. In addition, hypoxia can also serve as a chronic stressor to enhance seizure susceptibility by regulating neurosteroids and the HPA axis and activating the Wnt/β‐catenin pathway to regulate neurogenesis.

Due to the lack of research on chronic hypoxia and seizure susceptibility in the international academic community (research on the relationship between natural chronic hypobaric hypoxia and seizure susceptibility is even rarer), the relationship between chronic hypoxia and seizure susceptibility can only be studied and hypothesized by collecting fragmented data, as in‐depth research on the mechanism of the relationship between them is lacking. Our research group can only demonstrate our hypothesis at the broad and superficial levels of cytokines, proteins, and signaling pathways and cannot refine the arguments to support our hypothesis to a certain degree of specificity. Simultaneously, the lack of research on the specific mechanism and causal relationship between chronic hypoxia and seizure susceptibility also hinders the discovery of potential therapeutic targets and adjuvant therapy for epilepsy to some extent. In addition, the expression of some proteins and factors in various studies is controversial; for example, Wang, Q., et al. observed increased CX43 expression,^86^ whereas decreased expression was reported by Vezzani, A., et al. and Patel, D.C., et al.^11^, ^27^ Our team speculates that the reason for this paradox is the lack of unified and authoritative boundaries for chronic hypoxia; therefore, these changes cannot be accurately classified as the result of body compensation and decompensation. Although no authoritative limit has been established between compensation and decompensation, this review will discuss some of the research results that corroborate our hypothesis. At the same time, we are also interested in the conflicting aspects. Regarding the mechanism of chronic hypoxia and seizure susceptibility, our research group plans to conduct further research in the future to better test our hypothesis.

Because the Qinghai‐Tibet Plateau and other high‐altitude areas have typical environmental characteristics of chronic hypobaric hypoxia, the natural chronic hypobaric hypoxia environment at high altitudes may change the neuron excitability threshold in a manner similar to chronic hypoxia, thereby enhancing seizure susceptibility. At present, research on chronic hypobaric hypoxic environments and seizure susceptibility in international academic circles is lacking and most of the hypoxic conditions involved in a few similar studies were simulated by hypobaric oxygen chambers, the simulation effect is not completely consistent with a natural hypobaric hypoxic environment, and the research results may be partially controversial; In addition, despite the large differences between the living environment and genotypes of residents at high altitude and those living on the plain, no related studies have confirmed whether patients with epilepsy at high altitude have a tendency to present with drug resistance and whether the pharmacokinetics of antiepileptic drugs differs in those patients compared with the pharmacokinetics in the native population on the plain. Thus, the relationship between natural chronic hypobaric hypoxic environments and seizure susceptibility at high altitudes has considerable research prospects. Additionally, the relationship between chronic hypoxia and seizure susceptibility may provide many research directions in the field of neurology and high‐altitude medicine, including investigations of the functional sites and gene polymorphisms of EPAS1 and EGLN1, two key genes in the hypoxia pathway on the Qinghai‐Tibet Plateau, which may provide a direction for research on the mechanism underlying the relationship between chronic hypobaric hypoxia and seizure susceptibility. Furthermore, the key cytokine suppressor of cytokine signaling 3 (SOCS3) involved in the transformation of pro‐inflammatory M1 and antiinflammatory M2 microglia^54^ may provide a research direction for drug therapy targets or adjuvant therapy for patients with epilepsy at high altitude. In addition, it will be important to assess whether epileptic patients living at high altitude have a tendency to present with drug resistance and whether the pharmacokinetics of antiepileptic drugs differs in those patients compared with the pharmacokinetics in people living on the plain. Despite a lack of targeted research at present, studies on UGT gene polymorphisms and the antiepileptic pharmacokinetics of native Tibetans have been conducted on the Qinghai‐Tibet Plateau, and clinical and animal model studies on the effects of hypoxia inducible factor‐1α (HIF‐1 α) and multidrug resistance protein 1 (MDR‐1) on the function of antiepileptic drug transporters have also been carried out in the plain area.^87^ Therefore, we believe that with the progress of pharmacological technology and the continuous emergence of new antiepileptic drugs,^88^, ^89^ the relationship between chronic hypoxia and seizure susceptibility and whether there are individualized treatments that can significantly improve the efficacy of epileptic patients living at high altitude will experience exciting research progress.

Examining the relationship between chronic hypobaric hypoxic environments and seizure susceptibility in high‐altitude areas is important for exploring the regional characteristics of epilepsy; studying the potential diagnosis, prevention, and treatment of epilepsy; improving the diagnosis and treatment of epilepsy in plateau areas; and promoting the research and development of neurology, especially epilepsy, in the field of high‐altitude medicine.

## FUNDING INFORMATION

The research funding comes from the “Applied Basic Research of Qinghai Province 2022 Basic Research Program‐Study on chronic hypobaric hypoxia and seizure susceptibility at High altitude” (Grant # 2022‐ZJ‐761). We declare that any patient data involved in this manuscript has been ethically approved by relevant institutions.

## CONFLICT OF INTEREST

We clear that we have no conflict of interest.

## Data Availability

Data sharing does not apply to this article, and this research does not involve the analysis and innovation of new data.
